# Book Reviews

**Published:** 1853-10

**Authors:** 


### BIBLIOGRAPHICAL.

***Bull on the Maternal Management of Children.*** Philadelphia. Lindsay & Blakiston, 1853.

This valuable manual is now presented to us in a second edition, which, the preface tells us, is much improved, several of the chapters having been entirely re-written.

The book has been so long before the public that it is hardly necessary for us to say any thing about it. The importance to mothers, of such knowledge as is contained in it, cannot be overrated. So many children are destroyed by imprudence and ignorant, injudicious indulgence, that any treatise expounding to mothers the laws of the human frame and the hygienic management of this tender age cannot come amiss. Physiological knowledge of all kinds should be more widely diffused among the people. We should then have less quackery, less silly tampering with disease, a lower rate of mortality and more general physical comfort than we can now boast of. Nine-tenths of the mischief done by shallow books, and all the quackery that is in the world, can be traced to men's gross ignorance of the laws of their own existence.

These remarks are applicable with additional emphasis to the management of children. They are cast entirely upon our care, and we should be particularly zealous in faithfully discharging the duty which we have not wholly taken upon ourselves, which men have not thrust upon us, but which Almighty God, in his providence, has made incumbent upon us.

We, therefore, hail all such books as these as benefits to the world. There are many unintentional Herods in the nineteenth century, and many an Innocent's day which is not recorded in the calendar of any church. There is also but one way to put a stop to this squandering of young lives, to arrest this perpetual peregrination of the hateful little white hearse through our streets. It is by educating the masses in their duties to children, in the peculiar bodily habits of the little creatures, in the powerful influence of slight causes upon their tender frames.

This book, to a considerable extent, supplies this information; therefore we recommend it. There is, however, a strong temptation presented to the physician who gets up any thing of the kind, to write a treatise upon domestic medicine. The author of this little book has manifestly felt this temptation, and has yielded to it so far as to give a brief description of several childish ailments. We doubt the discreetness of such a course.

It is likely to torment an anxious, loving mother with many unnecessary disquietudes. It keeps her perpetually on the look out for symptoms of formidable diseases, in her little Eliza Jane or Augustus Frederick. It makes her fidgety and restless and fills her with dire apprehensions, and just in this proportion, it embarrasses the family physician, who cannot possibly keep pace with the mother's concern. The folly, however, of attempting to teach people to prescribe by the book is very generally avoided.

A few practical hints on poisons and their antidotes have been very judiciously introduced. Children have, as every observer of their habits knows, a terrible proclivity to put things of all sorts, from the spout of a tea kettle, full of boiling water, down to a painted toy, in their mouths, and it is not always possible for the most prudent and judicious *materfamilias* to keep noxious matters always out of the way. It is, therefore, very well for her to know what to do in cases of sudden emergency when medical aid cannot be immediately obtained.

*The Microscopist; or a Complete Manual on the Use of the Microscopes for Physicians, Students, and all lovers of Natural Science.* (Second edition, improved and enlarged.) By Joseph Wythes, M. D. Philadelphia. Lindsay & Blakiston, 1853.

The multiplication of works on the subject of microscopy is a cheering symptom. It shows that the minds of men are becoming gradually convinced of the great importance of this mode of interrogating nature. Originally a mere toy, the microscope has become a most valuable and indispensable means of research. By its aid, modern physiology is what it is. Even chemistry calls it to its assistance when it would unravel some problem of peculiar intricacy, or search for some deep and abstruse fact. The suspicion, with which the anatomists of the olden school regarded the instrument, is now gradually passing away, and a very general and increasing confidence in its teachings fills the mind of the profession.

The time is rapidly approaching when the microscope will be as necessary a portion of every physician's outfit as his pocket-case is now. Already it is a valuable means of diagnosis. No man in his senses, we presume, would pretend to treat an obscure case of urinary disease without first learning the microscopical characters of the urine.

In medico-legal investigations, it is also becoming important. In testing for arsenic, absolute certainty is now commonly attained by driving up and down the combustion tube the arsenical stain, till it is oxydated to arsenious acid, recognized by its octabedral crystals, when examined microscopically. In a recent trial, it became necessary to determine whether certain hairs on a hatchet were human or not. The microscope decided that they belonged to an animal, and so saved the life of a suspected murderer.

The little volume before us is an admirable hand-book for the beginner, who would commence the use of this valuable instrument. It contains a brief account of the optical principles on which the microscope is constructed, and of the different methods of applying those. Sketches and descriptions of compound simple and dissecting microscopes are given, while the various adjuncts are described and figured, and their uses pointed out.

Some pains is taken to inform the student how he may best procure microscopic objects, and under this, excellent directions are given for obtaining the rarer infusoria. Instructions are also given in mounting and preserving objects, and Goadby's excellent method is described somewhat in detail. The methods of making sections, minute injections, &c., are also mentioned, and the important subject of polarized light is not forgotten. Plans of polariscopes are given, and their attachment to the microscope is described.

In short, it is an excellent manual, and the numerous accurate cuts, with which it is filled, add greatly to its value.

---

*A Text Book of Anatomy and Guide in Dissections, for the use of Students of Medicine and Dental Surgery.* By W. R. Handy, M. D., Professor of Anatomy, in the Baltimore Dental College. Philadelphia, Lindsay & Blakiston, 1853.

Anatomical works are poured forth from the press in great abundance, and yet, a few favorites still keep possession of the field. A new candidate for public favor must, therefore, come before the world with something peculiar to recommend it, or it cannot claim the notice of a profession already so abundantly provided.

Professor Handy's work does possess these peculiarities. He has cast it in an entirely new and original mould. He has observed the same order in it which he follows in his lectures. He does not, for example, take up first the bones of the body and finish them, then the ligaments of the whole body, and finish them—then the muscles of the entire frame, and finish them, and so on through the catalogue, continually severing those things which nature has put together. On the contrary, he studies a part as it is, the bones of it first, of course, because they are its basis, then its ligaments and so on, till the whole part has been completely examined. In this manner, the student gets a clear and connected idea of what his teacher means that he shall learn, instead of being compelled to go over the whole field of anatomy and pick up the scattered facts, laboriously to combine the disjointed fragments in one entire and intelligible system, as Isis rambled over the world after the mangled and dispersed remains of the body of Osiris.

Dr. Handy, not only studies every individual part in all the relations of its elements, but he also dwells particularly upon the relations, anatomical and physiological of the part with neighboring organs and with the entire frame. Thus, a clear, connected, and natural system of teaching is arrived at, instead of the eminently artificial method commonly pursued.

He begins with what he calls the *alphabet of anatomy,* that is, the primary tissues of the body. Having studied these, he commences with the mouth, and follows the physiological course of the food in his demonstration. The extremities not having any direct relation to these functions, are described by themselves. We are satisfied, that a student will learn more that is valuable from this method, than from any other with which we are acquainted.

The book is copiously illustrated. Many of the cuts are entirely new in this country. We heartily commend it to both the medical and dental professions, as a thorough, faithful and physiological disquisition on anatomy.

*On the Loss of the Teeth, and on the Best Means of Restoring them; with a New and Improved Method of Fastening Loose Teeth.* By Thomas Howard, Surgeon Dentist to his Grace, the Archbishop of Canterbury. London.—(No date.)

We hope his Grace, the Archbishop of Canterbury, will endeavor to spare time from what devolves upon him daily, "the care of all the churches," to bestow a little attention upon the morals of his surgeon dentist. A few homilies to illustrate the maxim that "honesty is the best policy," would certainly be appropriate and might be beneficial. Considerably more than one-half of the surgeon dentist's book is taken word for word from Bell, without one syllable of acknowledgment, or the slightest mark that it came from any source but Mr. Howard's own brain.

Besides this capital defect of dishonesty, the book has a marvellously quackish savor. In one place, the dentist tells us, that he has "important reasons" for adopting a certain line of conduct. He hints that he has peculiar methods of operating, &c. In short, the book is an advertisement.

*The American Journal of Science and Arts, for September.*

This highly esteemed and oldest of the American Scientific Journals, so far from falling off, has gone on steadily increasing in interest and usefulness.

In the present number, we notice another accession to the able corps of editors who have hitherto had it in charge. Dr. Waldo I. Burnett, of

Boston, and Professor Louis Agassiz, of Cambridge, are now regular editors of the Journal. The former gentleman has become favorably known to the scientific world, by a number of valuable anatomical papers. The renown of the latter is world-wide, and he needs no introduction to any one who has the slightest smattering of natural history. Profoundly learned in every department of zoology and paleontology, he is the greatest living authority in ichthyology. He is engaged now in a work on the fishes of the United States, which will, undoubtedly, be the fullest and most satisfactory account of the subject that has ever appeared.

We know of no Journal which can boast of so distinguished a corps of editors. Every one of them is eminent in his particular department. Nor must we omit to mention the admirable Parisian correspondent of the Journal, M. J. Nicklés, whose letters contain an abstract of all the most important scientific news of the French capital.

Some idea may be formed of the variety of information contained in this Journal, by the following list of the contents of the present number:

On an isothermal oceanic chart, illustrating the geographical distribution of marine animals, by Dana; Contributions to mineralogy, by Genth; Hassler's experiments on the expansion of water, at various temperatures, by Alexander; Biography of Berzelius, by Rose; Artificial formation of minerals, by Marsoss; Probable number of the native Indian population of British America, by Lefroy; Constitution of some mineral species, by Hunt; Expenditure of heat in the hot air engine, by Barnard; Crystallized carbonate of lanthanum, by Blake; The normal of curvature, by Whitlock; Modification of the Ericsson engine, by Barnard; Parasitisn of comandra umbellata, by Gray; Reviews and abstracts in anatomy and physiology, by Burnett; Correspondence of M. J. Nicklés, containing researches in dying, pisciculture, carbonizing of wood by over-heated steam; Anæsthetic properties of lycoperdon, chloroformization, composition of water, extract of soils, photography, manufacture of sugar, &c.; together with a very full abstract of scientific intelligence in all departments.

*A Treatise on Operative Ophthalmic Surgery.* By H. Haynes Walton, Fellow of the Royal College of Surgeons, &c. First American from the First London Edition. Edited by S. Littell, M. D., Surgeon to Will's Hospital, &c. Philadelphia, Lindsay & Blakiston, 1853.

This is an excellent work on the subject. It is evidently the production of a man who has seen many cases of disease, and gathered from them much experience. He is thoroughly practical in his views, and in his mode of stating them. He does not confine himself to a mere operative surgery of the eye, but runs off now and then to the consideration of some points in the pathology and therapeutics of that delicate organ. His readers, however, by no means regret these little digressions, for they abound in valuable practical hints, and are characterized throughout by sound good sense, and thorough acquaintance with the subject.

The book is gotten up in admirable style. The paper is clear, white and heavy, and the type so clear that it is a luxury to read it. The cuts are by Gihon, and are beautifully executed. The mechanical management of the book leaves nothing to be desired.

---

*Chemistry and Metallurgy as Applied to the Study and Practice of Dental Surgery.* By A. Snowden Piggot, M. D., etc. Philadelphia. Lindsay & Blakiston, 1853.

In a former number of the Journal, the senior editor announced that Professor Piggot was engaged in writing a work on Chemistry and Metallurgy, as applied to Dentistry. He now has the pleasure of informing his readers that it will be published in a few days, by Messrs. Lindsay & Blakiston, Philadelphia. He has had an opportunity of examining most of the sheets, and from what he has seen, he has no hesitation in saying, that the work is admirably adapted to the wants of the dentist. It is well written, and has only to be read to be appreciated. Indeed, it fills a hiatus which has always existed in the literature of dental surgery.

The importance of a knowledge of chemistry and metallurgy to the dentist, is now so universally acknowledged, that no argument is necessary to establish the fact. This knowledge will be supplied in the fullest and most complete manner, by the work under consideration, and indispensable and valuable as it will prove to be to the dentist, it may be read with equal profit by the student and practitioner of general medicine.

The work is divided into four books. The *first* is an outline of organic chemistry. It contains an account, first, of the ultimate, and then of the proximate elements of the body; taking up first, the protein compounds, then the organic acids and bases in regular order. *Book second* contains an account of digestion, first in the stomach, and then in the intestines. It includes, of course, the chemistry of the gastric juice, the bile, the pancreatic fluid, the intestinal juice, the fæces and vomited matters.

*Book third* contains the chemistry of the mouth. This includes the the chemistry of saliva, healthy and morbid; the chemistry of the teeth, of mucus and of salivary calculus, as far as known.

*Book fourth* treats of the chemistry and metallurgy of metals and the earths used in the manufacture of porcelain teeth. It contains, first, an account of the various methods of applying heat, the construction of furnaces, crucibles, lutes, measurement of heat, and full tables of fuel, showing the economy of the different varieties. Secondly, the metals—bestowing particular attention on gold and silver. Very full tables of coins of these two metals are given, so that the mechanical dentist can be perfectly sure of the exact composition of his alloy. Thirdly, the earths and alkalies, the structure of porcelain, the method of preparing the materials and the mode of making and coloring artificial teeth.

The foregoing is but a very imperfect outline of the plan of the work. Time and want of space prevent us from entering into detail. H.

---

*Observations on the Disease and Loss of the Teeth, and the various means of supplying their Deficiency; and on Defects in the Palate, and their Treatment.* By ALFRED BARON JONES, Surgeon Dentist; Member of the Royal College of Surgeons, in England. 8vo, pp. 89. S. Highley & Son, London, 1853.

THE object of the above work seems to be, the instruction of the general reader, and has less the appearance of an advertisement than most popular treatises of this kind. The author is very well informed on the subjects on which he treats. His views are presented in a modest, but straight-forward manner, and he does not hold out the disgusting idea that his method of practice is peculiar to himself, and as a consequence, superior to that of his professional brethren. He frankly acknowledges, in the preface, his inability to throw any additional light upon the diseases of the teeth, and that the object he has in view, is, "to make the general reader familiar with the chief features of the most important diseases to which the teeth are liable."

The introduction is devoted to a brief description of the different substances which enter into the formation of a tooth, and the part which the teeth play in the first stage of digestion. Caries of the teeth next engages his attention, and here, he advocates the old doctrine of Paré, Hunter, Fox and Bell, that the structural alteration constituting the disease, is the result of inflammation of the dentine. The remedial indications of the disease are next noticed; after which, he treats successively, on "exostosis," "necrosis," "accidental injuries of the teeth," "effects of mercury," "gum-boil," "diffused abscess," "fall of the teeth in old age," "tartar," "diseases of the alveoli and gums," "artificial teeth," and "artificial palates."

Although we differ with the author in many of the opinions which he has adopted, still we cannot but admire the modest and dignified manner in which they are presented to the reader.

*Iowa Medical Journal.*

We have received the two first numbers of this periodical. It is a monthly. It is edited by the medical faculty of the Iowa University, and contains much instructive matter.

If, we mistake not, Keokuk, a new town on the frontiers of civilization, has now *two* medical periodicals. Baltimore, a century old, with two hundred thousand inhabitants, three hundred M. D's and half a dozen hospitals, has no medical journal, no medical association, no museum worth an hour's time to look through. Who is to blame?

*Peninsular Journal of Medicine and the Collateral Sciences.*

Another new candidate for professional favor and a promising one. Three numbers, those for July, August and September have reached us. E. Andrews, A. M., M. D., demonstrator of anatomy in the University of Michigan, is its editor. It is published at Ann Arbor. It is neatly gotten up as to its mechanical department, and the editor appears to be well qualified for his post. The numbers which we have received compare favorably with its contemporaries on our exchange list. We wish the editor success. He deserves it.

